# Student knowledge gains among first-time and repeat attendees of school-based asthma education program

**DOI:** 10.1186/s12890-023-02544-y

**Published:** 2023-07-10

**Authors:** Anna Volerman, Nicole Kappel, Ashu Tayal, Mary Rosenwinkel, Erica Salem, Lesli Vipond

**Affiliations:** 1 Department of Medicine, University of Chicago, 5841 S Maryland Ave, Chicago, IL 60637 United States of America; 2 Department of Pediatrics, University of Chicago, 5841 S Maryland Ave, Chicago, IL 60637 United States of America; 3 University of Chicago, Harris School of Public Policy, 1307 E 60th St, Chicago, IL 60637 United States of America; 4 Respiratory Health Association, 1440 W Washington Blvd, Chicago, IL 60607 United States of America

**Keywords:** Children, Pediatric, Youth, Iterative, Respiratory disease, Training

## Abstract

**Background:**

Because children spend much of their time in schools, schools can play an important role in asthma education for the one in 12 affected children in the United States. School-based asthma education programs are commonly repeated annually, however few studies have evaluated the impact of repeated participation in asthma education in school-based programs.

**Methods:**

This observational study evaluated the impact of Fight Asthma Now© (FAN), a school-based asthma education program for children in Illinois schools. Participants completed a survey at the start and end of the program, including demographics, prior asthma education, and 11 asthma knowledge questions (maximum knowledge score = 11).

**Results:**

Among 4,951 youth participating in the school-based asthma education program, mean age was 10.75 years. Approximately half were male and Black. Over half reported no prior asthma education (54.6%). At baseline, repeat attendees had significantly higher knowledge versus first-time attendees (mean: 7.45 versus 5.92; p < 0.001). After the program, both first-time and repeat attendees had significant knowledge improvements (first-time: mean = 5.92◊9.32; p < 0.001; repeat: mean = 7.45◊9.62; p < 0.001).

**Conclusions:**

School-based asthma education is effective for increasing asthma knowledge. Notably, repeated asthma education in school leads to incremental benefits for knowledge. Future studies are needed to understand the effects of repeated asthma education on morbidity.

## Background

Asthma affects one in 12 United States children and is associated with significant morbidity, including 767,000 emergency department visits and 13.8 million missed school days annually [1, 2]. Minority youth, specifically Black and Puerto Rican, are disproportionately impacted with 3–6 times higher rates of emergency department visits and hospitalizations [3–6].

Asthma management requires reducing triggers, monitoring symptoms, and using medications effectively. Education is a key part of self-management. National asthma guidelines recommend that education should occur at all points of care, including in clinical and community settings [7].

Schools are an opportune place for asthma education programs for children [8], as research shows that students learn better in an environment in which they are accustomed to learning [9]. Studies show school-based asthma education leads to improvements in knowledge among children, utilizing various knowledge questionnaires [10–13]. In addition, such programs are associated with improved asthma management skills, self-efficacy, symptoms, school attendance, and healthcare utilization among children [10–13].

While prior research demonstrates the positive effects of asthma education programs, few studies have examined the impact of repeated asthma education for school-aged children. One study examining the effects of repeated instruction shows it leads to increased likelihood that children have proper technique [14]. Whether the results are similar for general asthma education is unknown. Thus, to understand the potential incremental effects of asthma education programs for children, this study evaluated the impact of a school-based education program on knowledge among children participating in education for the first time versus those who have had prior education.

## Methods

### Study design

This observational study evaluated a school-based asthma education program—Fight Asthma Now© (FAN)—delivered in Illinois schools during the 2017–2020 school years. FAN has been previously shown to increase asthma knowledge among participants [15]; however whether its effects differ based on students’ prior participation in asthma education has not been previously evaluated. This study was deemed exempt from human subjects review.

### Intervention

The FAN program aims to help children with asthma identify and avoid triggers, manage episodes, and control their asthma long-term. FAN is administered by trained asthma educators at Respiratory Health Association to school-aged children. Content is standardized with delivery adapted based on student age and program length. In terms of student age, the content and activities are developed as a youth and a teen curriculum with age-appropriate language and topics utilized for each; for example, how to personally avoid smoking tobacco is included in the teen curriculum but not in the youth curriculum. In terms of program length, FAN is administered as three 60-minute sessions or four 45-minute sessions, based on each school’s schedule and preference.

### Participants

Participants in the FAN program included students who attended various school districts, including elementary, middle, and high schools, across Chicago, Suburban Cook County, and other areas within Illinois.

### Data collection

Just prior to and immediately after the education program, participants independently completed questionnaires focused on asthma knowledge, practices, and self-efficacy. In addition, students indicated whether they previously participated in asthma education and also provided their demographics (grade, gender, race/ethnicity). Eleven questions assessed knowledge about signs/symptoms, triggers, treatment, and disease course, while two questions assessed practices and one question assessed self-efficacy (multiple choice). Each question was scored as correct or incorrect. A knowledge score was created based on correctly answered questions with equal weight given to each question (maximum = 11).

### Data analysis

Children were classified into two groups based on their report of prior asthma education. Scores across groups were compared using the Mann-Whitney U test. Pre/post-program knowledge scores were compared using Wilcoxon Sign-Ranked test. Normality was checked using Shapiro-Wilk test. Changes in individual knowledge, practices, and self-efficacy questions were assessed using McNemar’s test. For multivariate analyses, ordinary least-squares regression was applied to account for covariates, including demographics (age, gender, race/ethnicity, location) and program (youth/teen, length, attendance). Analysis utilized Python 3.7.6. Significance was defined by p-value < 0.05.

## Results

Between 2017 and 2020, 4,951 students participated in FAN in 200 schools across 52 districts in Illinois. A total of 3,450 children completed pre- and post-questionnaires, with 2,566 completing all questions on both. Children’s mean age was 10.75 years (SD = 2.05). Half the children were male (49.8%, n = 1718/3450). Approximately half of participants were Black/African American (50.9%, n = 1755) and a smaller proportion were Hispanic (14.7%, n = 508). The majority were located in Chicago (68.1%, n = 2348) and participated in the youth program (74.0%, n = 2553). Over half the children reported no prior asthma education (54.6%, n = 1882).

Among all participants, the FAN program led to significantly increased asthma knowledge (mean (SD): pre = 6.43 (2.18), post = 9.42 (2.08); p < 0.001; n = 2566). In multivariate analyses, the results remained unchanged after accounting for covariates. Participants showed significant improvements in each of the 11 knowledge questions as well as the practices and self-efficacy questions (Table 1).

**Table 1:** Changes in asthma knowledge, practices, and self-efficacy with a school-based asthma education program for all participants and comparing first-time versus repeat asthma education participants

	All participants (N = 3450*)				First-time asthma education (N = 2324*)				Prior asthma education (N = 1126*)			
Knowledge	N	Pre	Post	p-value	N	Pre	Post	p-value	N	Pre	Post	p-value
1. Knowing your warning signs can help spot an asthma attack (episode).	3159	1852 (58.6%)	2857 (90.4%)	< 0.001	2110	1071 (50.8%)	1887 (89.4%)	< 0.001	1049	781 (74.5%)	970 (92.5%)	< 0.001
2. Being around people who are smoking can trigger asthma.	3133	2564 (81.8%)	2844 (90.8%)	< 0.001	2093	1675 (80.0%)	1902 (90.9%)	< 0.001	1040	889 (85.5%)	942 (90.6%)	< 0.001
3. A spacer helps get more medication into the lungs.	3060	1409 (46.0%)	2657 (86.8%)	< 0.001	2045	748 (36.6%)	1753 (85.7%)	< 0.001	1015	661 (65.1%)	904 (89.1%)	< 0.001
4. When using an asthma pump (inhaler), it is best to take in the medication with a slow breath.	3035	2485 (81.9%)	2664 (87.8%)	< 0.001	2025	1600 (79.0%)	1764 (87.1%)	< 0.001	1010	885 (87.6%)	900 (89.1%)	0.27
5. Quick-relief medication helps get rid of squeezing in the airways.	2999	1031 (34.4%)	2431 (81.1%)	< 0.001	1997	563 (28.2%)	1582 (79.2%)	< 0.001	1002	468 (46.7%)	849 (84.7%)	< 0.001
6. Quick-relief medication should help within 10 to 15 min.	2956	1357 (45.9%)	2441 (82.6%)	< 0.001	1967	808 (41.1%)	1614 (82.1%)	< 0.001	989	549 (55.5%)	827 (83.6%)	< 0.001
7. Controller medication helps reduce swelling and snot in the airways.	2898	1071 (37.0%)	2309 (79.7%)	< 0.001	1922	587 (30.5%)	1516 (78.9%)	< 0.001	976	484 (49.6%)	793 (81.3%)	< 0.001
8. If you have controller medication, you should take it everyday.	2861	1597 (55.8%)	2453 (85.7%)	< 0.001	1897	996 (52.5%)	1609 (84.8%)	< 0.001	964	601 (62.3%)	844 (87.6%)	< 0.001
9. You should try to stay calm during an asthma attack (episode).	2835	2431 (85.7%)	2551 (90.0%)	< 0.001	1883	1594 (84.7%)	1682 (89.3%)	< 0.001	952	837 (87.9%)	869 (91.3%)	0.0054
10. People with asthma can exercise.	2792	1996 (71.5%)	2231 (79.9%)	< 0.001	1859	1287 (69.2%)	1466 (78.9%)	< 0.001	933	709 (76.0%)	765 (82.0%)	< 0.001
11. Asthma is a lifelong illness.	2716	1051 (38.7%)	2131 (78.5%)	< 0.001	1803	654 (36.3%)	1416 (78.5%)	< 0.001	913	397 (43.5%)	715 (78.3%)	< 0.001
Practices												
12. Do you talk with adults at home about your asthma? / I will talk to the adults in my home about my asthma action plan and my asthma medication.	2680	1316 (49.1%)	2065 (77.1%)	< 0.001	1776	819 (46.1%)	1349 (76.0%)	< 0.001	904	497 (55.0%)	716 (79.2%)	< 0.001
13. Do you have your quick-relief inhaler with you at school?	2632	1212 (46.0%)	1557 (59.2%)	< 0.001	1745	744 (42.6%)	965 (55.3%)	< 0.001	887	468 (52.8%)	592 (66.7%)	< 0.001
Self-efficacy												
14. Do you feel comfortable carrying your quick-relief inhaler at school?	2566	1494 (58.2%)	1664 (64.8%)	< 0.001	1702	940 (55.2%)	1073 (63.0%)	< 0.001	864	554 (64.1%)	591 (68.4%)	0.02

*N represents number of children in this group who answered at least one question. A different number of children answered each question and percentages for each question are based on the number of children who answered that particular question correct on pre and post questionnaires

**Fig. 1:**
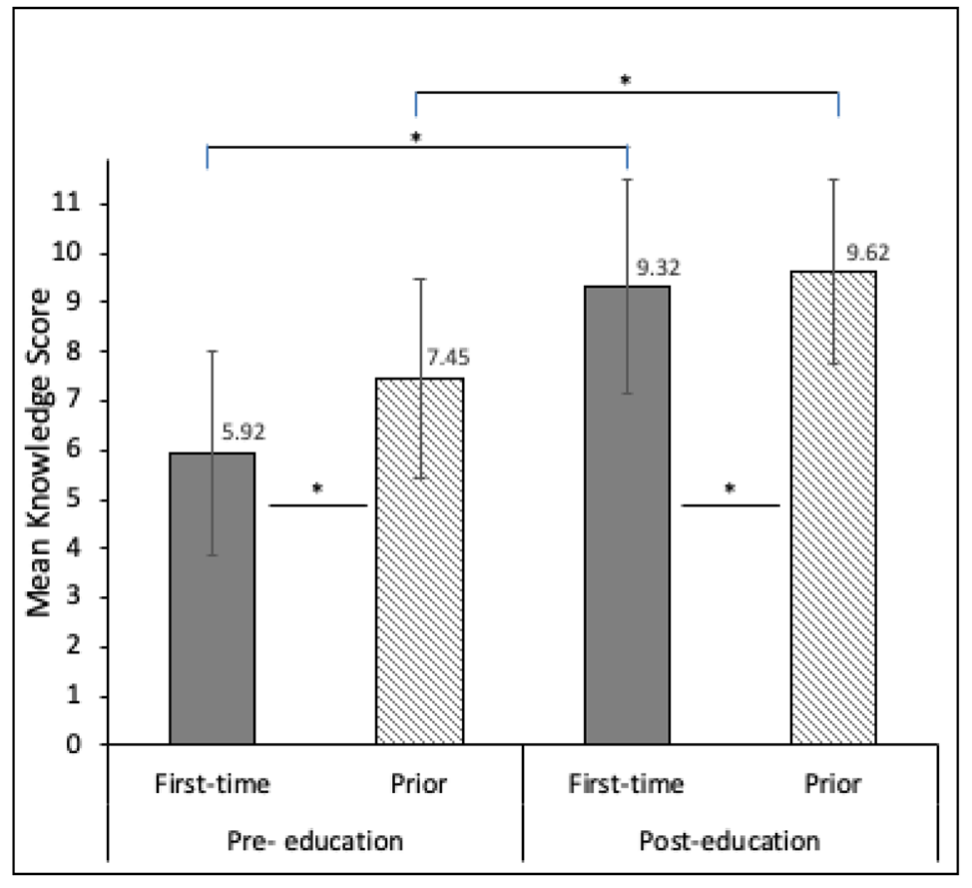
Children’s asthma knowledge before and after school-based asthma education program, comparing first-time participants versus those with prior asthma education. Students’ asthma knowledge was assessed before and after a school-based asthma education program, with a resultant knowledge score based on the number of correct responses out of 11. Baseline asthma knowledge was higher among prior asthma education participants as compared to children who reported receiving asthma education for the first time (mean (SD): first-time = 5.92 (2.08) versus prior = 7.45 (2.01); p < 0.001). Both first-time and prior asthma education participants showed significant knowledge gains as a result of the FAN program (mean scores: first-time = 5.92◊9.32, p < 0.001; prior = 7.45◊9.62, p < 0.001). Post-education, children with prior asthma education had significantly higher post-program knowledge scores than first time attendees (mean (SD): first-time = 9.32 (2.18), prior = 9.62 (1.86), p < 0.001)

At baseline, children who reported prior asthma education had higher knowledge compared to those participating in asthma education for the first time (mean (SD): repeat = 7.45 (2.01) vs. first-time = 5.92 (2.08); p < 0.001; Fig. 1). The FAN program led to significant increases in asthma knowledge for both first-time (mean score: pre = 5.92, post = 9.32; p < 0.001; n = 1702) and prior (mean score: pre = 7.45, post = 9.62; p < 0.001; n = 864) asthma education participants (Fig. 1). By question, first-time asthma education participants had significant improvement in all 11 knowledge questions compared to 10 questions among those with prior asthma education (Table 1). Both groups showed significant improvement in the practices and self-efficacy questions.

## Discussion

This study demonstrated that children who are first-time participants in asthma education and those with prior asthma education both had significant benefits as a result of a school-based asthma education program. While children with prior asthma education have higher baseline knowledge than first-time participants, the repeat attendees still showed significant knowledge gains at the end of the program. These findings align with prior research and builds upon the literature by demonstrating the value add of repeated asthma education [15–17].

In our study, FAN led to improved knowledge among students, consistent with prior school-based asthma education programs [15]. Children who reported receiving asthma education previously had higher baseline knowledge than those receiving such education for the first-time, as would be expected with effective asthma education. Importantly, both groups had significantly increased knowledge following the asthma education program. This finding suggests participation in asthma education leads to some knowledge gains even among children who receive repeat asthma education, in alignment with learning theory [18]. Repeated learning enhances memory retrieval and decreases forgetting of the subject matter, thus helping cement knowledge gains and enabling additive benefit to asthma education [19–21]. Notably, children with prior asthma education showed no improvement in one knowledge question (taking medication with slow breath), suggesting there may be a ceiling effect for some knowledge areas and the potential need to tailor educational content. Further, inhaler technique may differ from other knowledge areas given the importance of demonstration, practice, and reinforcement to cement technique.

This study has limited generalizability due to its focus on an educational program in one state with primarily minority children. Selection bias may be present as not all program participants completed questionnaires. Children’s reports of asthma education may be affected by recall bias and did not account for education type, timing, or quantity. This study’s results reflect asthma knowledge based a series of written questions and its clinically meaning or impact cannot be interpreted from these findings. Given that asthma education programs have been linked to better health and academic outcomes [22, 23], future studies should follow children longitudinally to understand the long-term impact of asthma education and determine optimal timing between repeated asthma education to impact knowledge, disease control, and healthcare utilization.

In summary, this study shows that asthma education leads to greater knowledge with significant incremental gains for children who previously received education. These results suggest the importance of repeated asthma education for children with potential to improve outcomes and reduce disparities.

## Conclusions

Given the findings of our study, schools should continue to, or begin to, implement school-based asthma education programs and leverage resources to create repeated programming so children can experience the incremental benefits of repeat asthma education. Specific consideration should be given to implementing such programs in schools that serve primarily minority and impoverished populations given the disproportionate effects of asthma within these communities. Specific consideration should be given to how to make in-depth asthma education available for students with asthma, for example as part of a special lunch or advisory group session. Schools should consider developing partnership with community-based organizations who lead such programs, as demonstrated in this study, to ensure the program is feasible to deliver. Further, longitudinal studies are necessary to understand the effects of repeated education on asthma morbidity in the long-term. Repeated asthma education programs in schools hold potential to increase knowledge, support better self-management, as well as potential to decrease disparities.

## Data Availability

The datasets used during the current study are available from the corresponding author on reasonable request.
